# Interpretability of a Deep Learning Based Approach for the Classification of Skin Lesions into Main Anatomic Body Sites

**DOI:** 10.3390/cancers13236048

**Published:** 2021-12-01

**Authors:** Joanna Jaworek-Korjakowska, Andrzej Brodzicki, Bill Cassidy, Connah Kendrick, Moi Hoon Yap

**Affiliations:** 1 Department of Automatic Control and Robotics, AGH University of Science and Technology, 30-059 Kraków, Poland; 2 Department of Computing and Mathematics, Manchester Metropolitan University, John Dalton Building, Chester Street, Manchester M1 5GD, UK; B.Cassidy@mmu.ac.uk (B.C.); Connah.Kendrick@mmu.ac.uk (C.K.); M.Yap@mmu.ac.uk (M.H.Y.)

**Keywords:** deep learning, transfer learning, malignant melanoma, skin cancer, convolutional neural networks, dermoscopy images

## Abstract

**Simple Summary:**

The detection of skin moles driven by current deep learning based approaches yields impressive results in the classification of malignant melanoma. It has been observed that the specific criteria for in situ and early invasive melanoma highly depend on the anatomic site of the body. To address this problem, we propose a deep learning architecture based framework to classify skin lesions into the three most important anatomic sites, including the face, trunk and extremities, and acral lesions. In this study, we take advantage of pretrained networks,we perform in depth analysis on database, architecture, and result regarding the effectiveness of the proposed framework. Experiments confirm the ability of the developed algorithms to classify skin lesions into the most important anatomical sites with 91.45% overall accuracy for the EfficientNetB0 architecture, which is a state-of-the-art result in this domain.

**Abstract:**

Over the past few decades, different clinical diagnostic algorithms have been proposed to diagnose malignant melanoma in its early stages. Furthermore, the detection of skin moles driven by current deep learning based approaches yields impressive results in the classification of malignant melanoma. However, in all these approaches, the researchers do not take into account the origin of the skin lesion. It has been observed that the specific criteria for in situ and early invasive melanoma highly depend on the anatomic site of the body. To address this problem, we propose a deep learning architecture based framework to classify skin lesions into the three most important anatomic sites, including the face, trunk and extremities, and acral lesions. In this study, we take advantage of pretrained networks, including VGG19, ResNet50, Xception, DenseNet121, and EfficientNetB0, to calculate the features with an adjusted and densely connected classifier. Furthermore, we perform in depth analysis on database, architecture, and result regarding the effectiveness of the proposed framework. Experiments confirm the ability of the developed algorithms to classify skin lesions into the most important anatomical sites with 91.45% overall accuracy for the EfficientNetB0 architecture, which is a state-of-the-art result in this domain.

## 1. Introduction

During the last few years it has been widely observed that malignant melanoma, the deadliest form of skin cancer, is becoming increasingly aggressive due to a combination of environment, genetics, and lifestyle. Most skin cancer cases are related to ultraviolet (UV) light damaging the DNA in skin cells. The statistics released by the American Cancer Society are alarming. It is projected that the number of new melanoma cases will increase by 5.8% in 2021 [1]. Furthermore, it is estimated that 207,390 cases of melanoma will be diagnosed in the U.S. in 2021, including 106,110 cases in situ (noninvasive) and 101,280 invasive cases, penetrating the epidermis into the skin’s second layer. The staggering rates show that global action including redefining of medical diagnostic algorithms and early diagnosis and novel treatment methods are needed in order to achieve control of melanoma mortality rate reduction and prevention of severe cases.

The most widely used medical diagnostic algorithms include pattern analysis, the ABCD rule of dermoscopy, and the so-called seven-point checklist, which are based on a critical, simultaneous assessment of so-called dermoscopic criteria. Argenziano et al. confirmed that diagnostic algorithms improved the rate of diagnosing pigmented skin lesions by 10–30% [2]. However, due to the lack of access to large datasets, the algorithms have not been adapted and adjusted for skin changes depending on the place of origin. It has been observed that the criteria for melanoma in situ and early invasive melanoma is highly dependant on the anatomic site of the lesion origin for the three main anatomic sites including (1) trunk with extremities, (2) face, and (3) palms and soles (acral lesions) [2].

The currently proposed computer-aided methods have been designed to extract and calculate significant features based on the entire dermoscopic dataset and distinguish between benign and malignant skin lesions. However, when dealing with melanoma originating in different parts of the body, no detailed research studies have been published so far.

This study aims to perform an experimental study in order to determine the ability of algorithms to recognize the anatomical site based only on dermoscopic images. We propose a novel framework for distinguishing between pigmented skin lesions based on site-specific dermoscopic characteristics of skin lesions originating in different anatomic sites of the body. We achieve this goal with the application of pretrained convolutional neural networks (CNN), their interpretability, and connection to the domain knowledge.

The information about the body location of the analysed skin lesion can be exploited as an additional channel in the CNN based architecture or as a parameter determining the selection of the next step of the classification system in the two-stage decision making process. Furthermore, it can be very beneficial to add such an algorithm to prove whether the assigned location seems to be correct or not. During a body examination, several lesions are analyzed for one patient (sometimes even more than 20). There are systems that require marking the place of origin right after taking the medical image and those that require adding anatomical site annotation at a later stage, after registering all skin moles. It seems that the automatic checking of the origin of the skin mole can be valuable and result in more accurate detection of malignant melanoma. Moreover, automatic information about the place of origin of a section for the histopathological examination may also be helpful in assessing the lesion if it has not been provided at an earlier stage.

The novelty of this work can be summarized as follows:

- We present a new approach based on the adjusted pretrained EfficientNetB0 network architecture for the classification of skin moles into anatomic sites of the body, which confirms that melanoma-specific criteria occurring in particular sites enable differentiation between them.
- We compare the outcomes of state-of-the-art pretrained models including VGG19, ResNet50, Xception, DenseNet121, and EfficientNetB0. We visualize the feature distribution extracted by each architecture.
- We propose a new approach for model interpretability based on comparing Grad-CAM heatmaps with the segmentation ground-truth for assessing the skin lesion classification process.
- We compare and estimate the correlation between feature importance and domain knowledge.

### 1.1. Motivation and Clinical Definition

The main motivation to undertake this research is the difficulty observed in correct visual assessment of dermoscopic images by inexperienced dermatologists who typically achieve sensitivity and specificity at around 62–63% [2]. Furthermore, the varied appearance and relevance of melanoma-specific criteria present in skin lesions originating in different anatomic sites can cause serious problems during visual assessment. In recent years, the diagnostic criteria have been proposed and tested by several authors [3,4,5].

**Figure 1:**
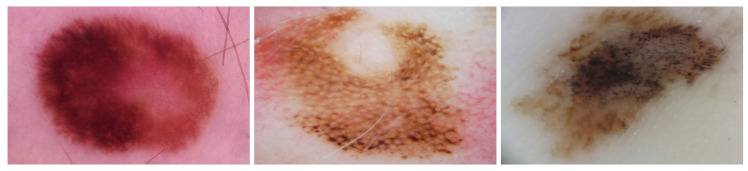
Dermoscopic images of in situ or early invasive melanomas presenting different dermoscopic features according to the anatomic site: (**left**) melanoma on the leg characterized by an atypical pigment network and irregular streaks, (**middle**) melanoma on the face characterized by reticular pattern, and (**right**) acral melanoma characterized by parallel-ridge pattern and irregular pigmentation.

In Table 1, we present the most important melanoma-specific criteria for melanoma in situ and early invasive melanoma, which contribute to the diagnosis where the frequency of the criteria is >70% [2]. For thick and advanced melanomas, the preformed anatomic structures responsible for the site-specific dermoscopic appearance are already destroyed and are independent of the various sites. Table 1 shows the dermoscopic criteria that are commonly observed in skin lesions heavily dependent on the anatomic site of the body. For trunk and extremities, the more common melanoma-specific criteria include multi-component pattern and atypical pigment networks, in contrast to the face where reticular patterns and atypical pigment pseudonetworks are always present. For skin moles located on palms and soles, the presence of parallel-ridge patterns is considered highly important (Figure 1).

### 1.2. Related Studies

In recent years, numerous clinical decision-support systems and computer-aided diagnostic systems have emerged for the automatic diagnosis of melanocytic lesions. These systems implement deep neural networks capable of classification of malignant and benign lesions. To the best of our knowledge, this study represents the first attempt to classify skin lesions into three main anatomic sites and proposes a new benchmark for the classification of skin lesions dedicated separately for each subtype. These subtypes include trunk with extremities, face, and palms and soles (acral lesions). However, we present the most recent studies concerning the classification of skin lesions from the respective anatomical regions.

Yu et al. [6] created a VGG-16 network trained on dermoscopic images of hands and feet consisting of acral melanoma and benign nevi confirmed by histopathological examination. This binary classification network demonstrated true positive, true negative, and area-under-the-curve measures similar to expert dermatologists and was able to outperform junior physicians. However, the dataset used was comparatively small—a total of 724 dermoscopic images consisting of 350 images of acral melanoma and 374 images of benign nevi.

Le et al. [7] devised a ResNet50 ensemble network for the classification of seven skin lesion types, including melanoma. This network used class weighing with a focal loss function to address the class imbalance of the HAM10000 dataset used for training their network. They achieved top-1, top-2, and top-3 accuracy, 93%, 97%, and 99%, respectively. This work observed that the gradual removal of the surrounding skin using U-Net segmentation resulted in increasingly reduced network performance. This suggests that the skin textures surrounding lesions are an important contributing factor to network accuracy and may be a vital pointer to any future networks trained to identify lesions by anatomical site.

Winkler et al. [8] investigated the diagnostic performance of FotoFinder Moleanalyzer Pro [9]—a commercially available CNN. Their experiment involved a binary classification (malignant/benign) for different melanoma localizations and subtypes using six dermoscopic datasets, which included melanomas of acral skin. This study noted that for acral melanomas, the system showed reduced sensitivity at high specificity.

Han et al. [10] created a localization network comprising a blob detector, a fine-image selector, and disease classifier. Their heterogeneous dataset comprised unprocessed photographs of malignant and benign lesions, which included lesions located on the head and neck. This study noted the limitations of using only dermoscopic images to train deep learning models that would be used in real-world settings due to the large number of complex shapes present on the human body, including acne and acne scars.

González-Cruz et al. [11] also noted limitations of datasets used in deep learning research for melanoma detection. They analyzed a dataset of 2849 high quality dermoscopic images of skin tumours to determine suitability for machine learning analysis. Their findings indicate that a large number of tumours located on the head, neck (76.8%), and trunk (>53.1%) had potential exclusion criteria due to absence of normal surrounding skin and pigmentation.

## 2. Database Specification

Nowadays, the most widely used dermoscopic skin lesion image database is the fourth ISIC dataset released by [12,13,14].

The ISIC 2019 dataset contains 33,569 dermoscopic images with patient metadata for the training set, indicating anatomical site of 22,700 lesions from a total of 25,331. Part of the ISIC 2019 dataset comprises the HAM10000 dataset, constituting the majority of dermoscopic images that are associated with the anatomical site. HAM10000 has been released by [12] and contains 11,526 dermoscopic images with metadata indicating anatomical site for 9781 lesions in the training set. The dataset contains 7222 dermoscopic images representing skin lesions originating in three different anatomic sites of the body including 6225 trunk/extremities, 702 face/head, and 295 acral lesions.

Due to highly imbalanced class composition, we augmented acral and face lesions by randomly applying image transformations such as rotation, sheer, and zoom. Each acral image was augmented 21 times, and each face image was augmented nine times, creating 6195 and 6318 artificial images, respectively. Augmentation was completed after we split the data into train, validation, and test subsets to avoid leaking information between subsets.

### Data Visualization

**Figure 2:**
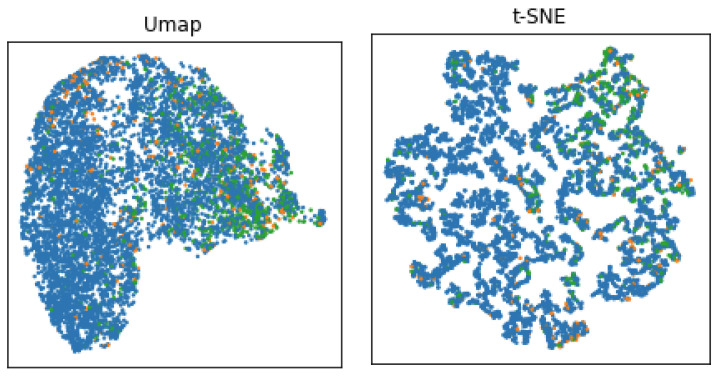
Visualization of HAM10000 dataset distribution on three anatomic sites with UMAP and t-SNE data transformation. The blue dots represent trunk and extremities, orange dots represent acral, and green dots represent face and head skin lesions.

In order to understand the distribution of the dataset, we visualize the data distribution of HAM10000 using two-dimensional reduction techniques—Uniform Manifold Approximation and Projection (UMAP) [15] and the t-distributed Stochastic Neighbor Embedding technique (t-SNE) [16]. UMAP is a manifold learning technique for dimension reduction, and t-SNE is an unsupervised method that maps similarities between high-dimensional data into a probability distribution in such a manner that similar objects have a higher probability, minimizing the Kullback–Leibler divergence between the two distributions [16]. Figure 2 shows the visualisation of dataset distribution using UMAP and t-SNE and the relationship between anatomical sites of the body.

We observe that skin lesions originating on the face form clusters of green dots while acral cases show irregular distribution. In order to analyze the datasets, we have calculated statistical metrics for (IntraC) intra-class and (InterC) inter-class ratio together with the ratio between InterC and IntraC (Ratio), computed using the Euclidean distance. Moreover, we analyzed the Silhouette Coefficient (*Silh.*), which is given by [17] as follows:

(1) $$Silh.=\frac{b-a}{max(a,b)}$$

where *a* is the mean distance between a sample and all other points in the same class, and *b* is the mean distance between a sample and all other points in the next nearest cluster. The best value is 1 and the worst value is −1. Values near zero indicate overlapping clusters. Another relevant metric is the Calinski–Harabasz (CH) index, also known as Variance Ratio Criterion, and it represents the ratio of the sum of between-cluster dispersion and of within-cluster dispersion for all clusters within the dataset. The dispersion is given as the sum of distances squared [18]. Additionally, the Davies–Bouldin index has been calculated, which signifies the average similarity between clusters as a measure that compares the distance between clusters with the size of the clusters themselves and is defined as follows [19]:

(2) $$DB=\frac{1}{k}\sum_{i=1}^{k}\max_{i\neq j}R_{ij}$$

where

(3) $$R_{ij}=\frac{s_{i}+s_{j}}{d_{ij}}$$

and $s_{i}$ is the average distance between each point of cluster *i* and the centroid of that cluster, $d_{ij}$ is the distance between cluster centroids, and *k* is the number of clusters.

In Table 2, we present the statistical analysis of the HAM10000 dataset in terms of the distribution of lesions regarding the anatomical site of the body. We observe that the complexity of the underlying classification task is very high and that regular machine learning algorithms will not be able to provide sufficient results. A high intra-class distance value indicates that cases are widely distributed in the space and hardly separable. However, as the inter-class distance is higher, measuring the difference between two classes, it indicates the possibility of separating the data into anatomical sites.

**Figure 3:**
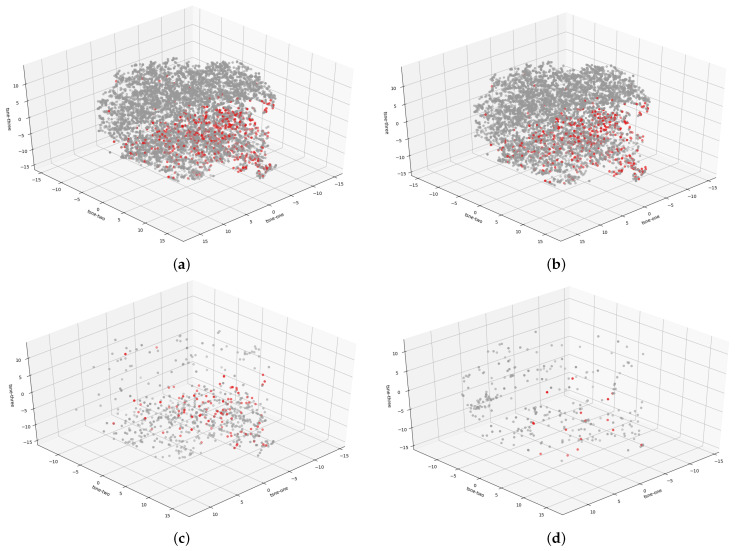
Interpretation of the 3D t-SNE plot visualization of the HAM10000 dataset where the red dots indicate melanoma cases while gray dots represent benign lesions. The following figures present the distribution of malignant and benign cases for the (**a**) entire dataset, (**b**) trunk-extremities dataset, (**c**) face-head dataset, and (**d**) acral dataset.

Furthermore, Figure 3 presents the distribution of melanocytic lesions within the disjoint dataset into the anatomic site. We observe that the red dots, representing malignant lesions, form areas and shapes that will be easier to separate than in the entire dataset. This is further confirmed by Table 2, which shows that the Silh. score and DB values indicate a better partition between trunk/extremities and the entire dataset.

## 3. Method

### 3.1. Determination of the Anatomic Site of the Skin Lesion

**Figure 4:**
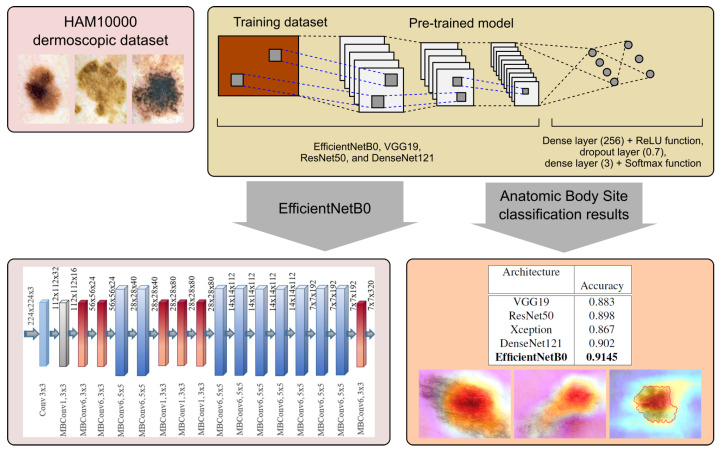
The streamline of our proposed framework. We use pretrained deep learning models including VGG19, ResNet50, DenseNet121, and EfficientNetB0 for feature extraction on the HAM10000 dataset. We employ the extracted features to conduct the multi-class classification task. Finally, we perform model evaluation and interpretation based on the heatmaps.

An overview of our method is illustrated in Figure 4. We reuse deep CNN models pretrained on the ImageNet dataset for feature extraction using the prepared HAM10000 dataset for the classification of skin lesions into anatomic sites of the body. We adjust the classifier, which has a three layer structure. As a result, we generate classification outcomes for the most widely used pretrained networks and analyze them. We further employ the Grad-CAM algorithm to generate heatmaps in order to conduct multi-task learning model interpretability.

### 3.2. Separability Analysis Using Deep Learning

**Figure 5:**
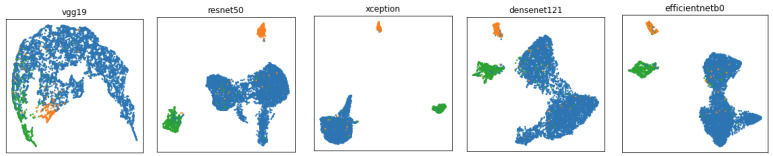
Visualization of full dataset feature distributions extracted with VGG19, ResNet50, Xception, DenseNet121, and EfficientNetB0. These graphs visually illustrate the separability of three anatomic sites.

We analyze the capability of the existing deep learning frameworks in discriminating three anatomic sites (trunk and extremities, acral, and face/head). This analysis will inform the design of our proposed method. For this preliminary analysis, we trained the models for 25 epochs without pretrained models and without data augmentation. Figure 5 presents the visualization of the data distribution by each network. The statistical metrics presented in Table 3 confirm that the three anatomic sites are separable and create clusters, where the intra-class values are lower and inter-class values are much higher. We observed that all of the implemented pretrained networks achieved high values for the CH index, which indicates huge potential in obtaining good results for the classification task. Considering the small size and imbalanced nature of the dataset, we propose several strategies to overcome these challenges in the following section.

### 3.3. Pretrained Model Based Architecture

Due to our limited and imbalanced dataset we take advantage of the transfer learning concept which indicates the effectiveness of reusing pretrained CNN architectures to extract the feature representation. There are several strategies of performing transfer learning including fine-tuning and feature extraction. However, due to our problem specification we propose a CNN based architecture which consists of a pretrained convolutional base and an adjusted classifier. We tested several state-of-the-art architectures including VGG19 [20], ResNet50 [21], DenseNet121 [22] and the latest EfficientNetB0 [23]. EfficientNet models which have been introduced in 2019 by Tan et al. are based on the inverted bottleneck residual blocks of MobileNetV2 and squeeze-and-excitation blocks. They use a compounding scaling method which scales width, depth, and resolution together instead of scaling only one model attribute. The EfficientNetB0 architecture has been proposed by a multi-objective neural architecture search which optimizes both accuracy and floating-point operations. Furthermore, a new activation function, Swish, has been proposed which shows superior performance for deeper networks. Swish is a multiplication of a linear and a sigmoid activation [23]:

(4) Swish(x)=x*sigmoid(x)

On top of the base, we have adjusted a fully connected classifier that contains the following layers: dense layer with 256 neurons and ReLU activation function, additional dropout layer which randomly sets input units to 0 with frequency of rate 0.7 at each step during training time as a regularization technique for reducing overfitting [24]. The architecture closes with a dense layer with the number of neurons corresponding to the number of classes and Softmax activation function for the predict a multinomial probability distribution.

### 3.4. Deep Learning Architecture Training

For each of the pretrained architectures including VGG19, ResNet50, Xception, DenseNet121, and EfficientNetB0, we deployed randomized search (RandomizedSearchCV) for optimizing hyperparameters including number of epochs, optimizer, and batch size [25]. The algorithm selected 20 random sets of parameters from an established range, maintaining an equal distance in a search space. We tested batch size and number of epochs from ranges $batch_{size}=8,16,\cdots ,512$ and $nb_{epochs}=5,10,\cdots ,50$, respectively, and tested several optimizers including RMSprop, SGD, Adadelta, Adam, and Adamax. The learning rate was left at default, as it greatly varies between different optimizers. Hyperparameter optimization was performed using 3-fold cross-validation on a training set. By training our model repeatedly with different parameters from this grid, we were able to select a more narrow area of parameters. Then, we used Grid Search, which performs an exhaustive search on all different hyperparameter combinations, for a much smaller range of parameters. Finally we empirically tuned those numbers further by analysing the model’s behaviour on a separate validation set and, for example, stopping the training earlier to avoid overfitting. After deciding the final set of parameters for each network architecture, we trained the models again, five times each, this time also checking the model’s performance on a completely separate test set. Achieved results for each training were averaged. Final parameters and results are presented in Table 4.

**Figure 6:**
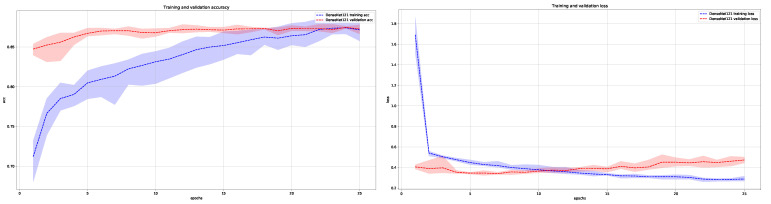
Average training and validation accuracy (**left**) and loss (**right**) during training of DenseNet121 for five times with maximal and minimal deviation areas marked in color (blue for training and red for validation).

**Figure 7:**
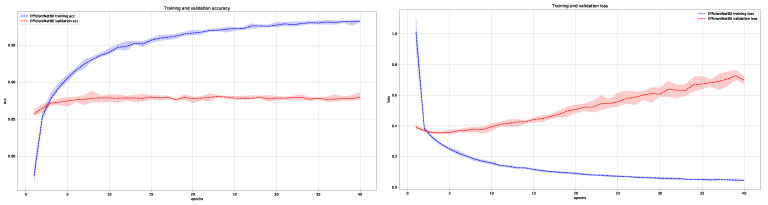
Average training and validation accuracy (**left**) and loss (**right**) during training of EfficientNetB0 for five times with maximal and minimal deviation areas marked in color (blue for training and red for validation).

In Figure 6 and Figure 7, we show the average training and validation accuracy for DenseNet121 and EfficientNetB0 architectures, which are the top two performers, and achieved the highest score in classifying skin lesions into the three main anatomical sites.

## 4. Experimental Results

### 4.1. Statistical Metrics

We compare the ability of state-of-the-art algorithms in classifying dermoscopic images of skin moles into three main anatomic sites of the body, including trunk/extremities, face/head, and acral lesions on five state-of-the-art deep learning networks, i.e., VGG19, ResNet50, Xception, DenseNet121, and the latest EffcientNetB0. The evaluation of the implemented and optimized architectures has been performed by using 20% of the dataset. The test results have been calculated five times and averaged.

The following performance metrics have been calculated based on the confusion matrix: accuracy (*ACC*), precision (*PPV*, positive predicted value), recall (*SE*, Sensitivity), and F1-score, where we specify the following: *TP* (true positive), *FN* (false negative), *TN* (true negative), and *FP* (false positive) values.

Accuracy, which measures statistical bias and systematic error, refers to the closeness of the measurements to a specific value and can be expressed as follows.

(5) $$ACC=\frac{TP+TN}{TP+FN+FP+TN}$$

Precision refers to random errors, and it is a measure of statistical variability, which describes the closeness of the measurements to each other and can be written as follows.

(6) $$PPV=\frac{TP}{TP+FP}$$

Recall measures the proportion of actual positives that are correctly identified as such and is defined as follows.

(7) $$SE=\frac{TP}{TP+FN}$$

F1-score (also F-score) considers both the precision and the recall of the test to compute the final score and is a measure of the test’s accuracy. The F-score can be expressed as follows.

(8) $$F_{1}=\frac{2\cdot PPV\cdot SE}{PPV+SE}$$

### 4.2. Effectiveness of the Proposed Framework

From the results presented in Table 4, we can conclude that all models were able to correctly recognize anatomic sites with high accuracy. Table 4 presents the evaluation metrics for each network architecture for the best set of training hyperparameters (optimised using grid search method described in Section 3.4). EfficientNetB0 achieved 91.45% accuracy and 91.5% F1-score, precision, and recall, which were the best results when trained with 45 epochs, batch size of 128, and the Adamax optimizer [26]. High precision and recall indicate the overall good performance of the model, with no visible biases. From the group of other architectures, only DenseNet121 managed to overcome the barrier of 90%, with others being slightly worse.

In addition to mentioned statistical metrics, we also assessed the effectiveness of the proposed framework using various visualisation and interpretability techniques, including our own metric, which we further describe in the next section.

### 4.3. Model Interpretability Based on Heatmaps Analysis

In order to improve model explainability, we used the Grad-CAM visualization algorithm [27], which creates a heatmap that shows which parts of the input image contributed the most to the classification. Furthermore, we performed an overlapping of the heatmaps with the segmentation ground-truth provided by Tschandl et al. [28].

**Figure 8:**
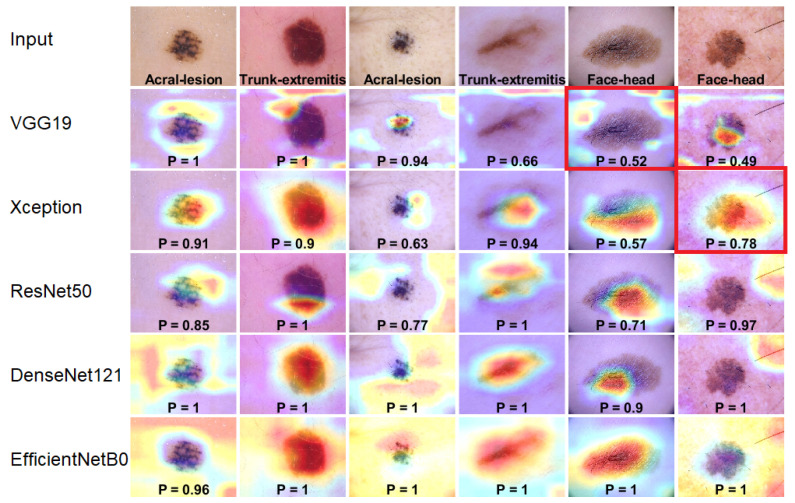
Grad-CAM visualization results. We compare the visualization results for each integrated pre-trained network based on the classification of skin lesion into the anatomic site of the body. The input image is shown on the top, and *P* denotes the Softmax score.

In Figure 8, we present two examples for each anatomic site with their corresponding heatmaps for pretrained architectures. Regions on which the network focuses are marked in bright colors superimposed on the input image. From these images, we can draw several conclusions. Firstly, we observe that the proposed architectures do not always concentrate on the region of interest. For VGG19 and ResNet50, the classification is mostly based on the surrounding area resulting in a low Softmax score (*p* value) within the range of 0.4–0.7, while DenseNet121 and EfficientNetB0 calculated the final score based on the skin lesion area and achieved the highest *p* value close to one. Furthermore, EfficientNetB0, which achieves the best results, tends to take very large areas into consideration instead of focusing on a single area.

Acral cases were found to be mostly classified based on the background of the skin, which is connected to the papillary pattern occurring in palms and soles. Trunk and face skin lesion images are classified based on the area of the lesion. These results provide strong evidence of the importance of differentiating between skin lesions originating in different parts of the body.

**Figure 9:**
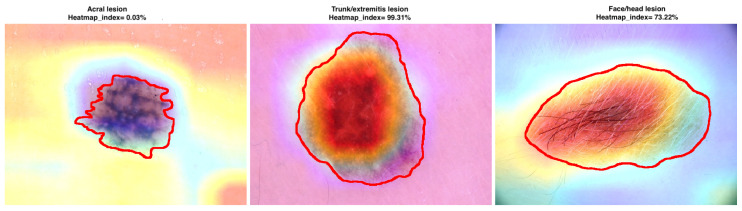
Examples of skin lesions originating in different anatomic sites with the corresponding heatmap created by the EfficientNetB0 model and segmentation ground-truth marked with red color. $Heatmap_{index}$ indicates to what extent the classification is based on the area of the skin lesion.

Moreover, we have proposed and calculated an overlapping index that compares the areas between heatmaps and segmentation ground-truth images. It confirms to what extent the classification is based on the area of the skin lesion. The $Heatmap_{index}$ is defined as the sum of intensity pixels in the heatmap within the segmentation area divided by the sum of all pixels in the heatmap. The formula is given by the following:

(9) $$Heatmap_{index}=\frac{\sum_{\left(x,y)\epsilon |H\cap S\right|}H\left(x,y\right)}{\sum_{(x,y)\epsilon H}H\left(x,y\right)}\cdot 100\%$$

where *H* is the heatmap image, and *S* is the binary segmentation mask. Based on the proposed overlap coefficient, we can observe (see Figure 9) and confirm that the classification has been mostly performed based on the skin lesion area for skin lesions originating in trunk/extremities and face, while the acral lesions have been classified based on the surroundings.

### 4.4. Software and Hardware

This research study was conducted using Python 3.7 programming language with Keras 2.3 [29] and scikit-learn [30] libraries. The models were trained on a NVIDIA RTX 2070 Super GPU (8 GB) with 48 GB RAM and Intel i7 Processor.

## 5. Conclusions

In this study, we developed a deep learning architecture based framework capable of skin lesion classification of the three main anatomical sites trained on the HAM10000 dataset. The network was shown to have high accuracy (>91%) in the classification of face, trunk and extremities, and acral anatomical regions. Furthermore, a heatmap analysis was used to determine locations on dermoscopic images in which the network based its classification on. The resulting architecture shows that features within dermoscopic images can be used to determine anatomical locations of skin lesions.

## Figures and Tables

**Table 1:** Common melanoma-specific criteria for melanoma in situ and invasive melanoma detection according to the anatomic site of the body based on [2].

Anatomic Site	Criterion	Description	Frequency
Trunk, extremities	Multicomponent pattern	Combination of few dermoscopic structures	Very common
	Atypical pigment network	Irregular brown to black network	Very common
	Irregular dots and globules	Black or brown oval structures	Common
	Irregular streaks	Irregular linear structures	Common
	Irregular pigmentation	Pigmented areas with irregular size and distribution	Common
Face	Reticular pattern	Diffuse pigmentation of the erpidermis or papillary dermis	Always present
	Atypical pigment pseudonetwork	Advanced morphological structures by melanoma progression	Always present
Palms and soles	Parrallel-ridge pattern	Pigmentation along the cristae superficiales	Very common
	Irregular dots/globules	Black or brown oval structures	Common
	Irregular pigmentation	Pigmented areas with irregular size and distribution	Common

**Table 2:** Statistical analysis of the dataset including calculations for the entire HAM10000 dataset regarding malignant and benign lesions as well as distribution in the acral and non-acral subsets.

Anatomic Site	# Total Nb.	#Melanoma Cases	Metrics				
			IntraC	InterC	Silh	CH	DB
Acral lesions	295	16	331.30	360.62	0.12	3.87	3.96
Face/head	702	102	317.65	324.655	0.03	6.75	6.84
Trunk/extremities	6225	490	338.26	356.12	0.12	90.27	4.57
HAM10000	7222	608	338.40	353.92	0.10	95.21	4.88

**Table 3:** Statistical analysis on the separability of three anatomic sites (trunk, acral, and face/head) of the HAM10000 dataset using UMAP visualization on deep learning methods.

Method	Metrics						
	Intra (Trunk)	Intra (Acral)	Intra (Head)	Inter-Class	Silhoutte	CH	DB
Input	4.2582	4.2815	3.3802	4.5036	−0.0016	251.8074	3.8125
VGG19	7.0763	3.4522	3.7104	7.5375	0.0658	822.4723	1.5865
ResNet50	3.9671	1.4387	1.7728	9.9075	0.4192	3463.3257	0.5819
Xception	3.0442	3.6002	3.0653	17.6211	0.8052	15,137.3670	0.3198
DenseNet121	4.2608	1.9341	1.7729	6.5770	0.3685	1919.5011	0.7422
EffcientNetB0	4.4417	2.2625	2.0617	9.1643	0.5488	3458.3440	0.6102

**Table 4:** Anatomic body site classification results for different neural network architectures with optimal set of parameters for each network and input images resized to 224 × 224.

Architecture	Optimal Training Hyperparameters			Metrics			
	Optimizer	Batch Size	Epochs	Accuracy	Precision	Recall	F1
VGG19	SGD	64	25	0.883	0.89	0.89	0.89
ResNet50	SGD	32	50	0.898	0.90	0.90	0.90
Xception	Adadelta	128	35	0.867	0.87	0.87	0.86
DenseNet121	Adam	64	25	0.902	0.90	0.90	0.90
**EfficientNetB0**	**Adamax**	**128**	**45**	**0.9145**	**0.915**	**0.915**	**0.915**

## Data Availability

The data presented in this study are available in this article.
